# The romantic loneliness scale (RomLon scale): the development and validation of a novel and brief measure to assess loneliness stemming from the absence of romance in dating, hookups, and marital relationships

**DOI:** 10.1186/s40359-025-03208-8

**Published:** 2025-08-11

**Authors:** Waqar Husain, Muhammad Ahmad Husain, Farrukh Ijaz, Javeria Farrukh, Fatima Batool, Nimra Tahir, Khaled Trabelsi, Achraf Ammar, Haitham Jahrami

**Affiliations:** 1 Department of Humanities, COMSATS University Islamabad, Islamabad Campus, Park Road, Islamabad, Pakistan; 2 Research laboratory Education, Motricité, Sport et Santé, EM2S, LR19JS01, High Institute of Sport and Physical Education of Sfax, University of Sfax, Santé, High Tunisia; 3 Department of Movement Sciences and Sports Training, School of Sport Science, The University of Jordan, Amman, Jordan; 4 Department of Training and Movement Science, Institute of Sport Science, Johannes Gutenberg-University Mainz, Mainz, Germany; 5 Research Laboratory, Molecular Bases of Human Pathology, LR19ES13, Faculty of Medicine of Sfax, University of Sfax, Sfax, 3000 Tunisia; 6 Department of Psychiatry, College of Medicine and Health Sciences, Arabian Gulf University, Manama, Bahrain; 7 Government Hospitals, Manama, Bahrain

**Keywords:** Romantic loneliness, Sexual distress, Hopelessness, Psychosocial health, Measurement and assessment

## Abstract

**Background:**

Despite extensive research on general loneliness, existing scales overlook romantic loneliness as an independent construct, hindering the understanding of its psychological impact. The present study aimed to develop and validate a concise instrument to assess romantic loneliness: the Romantic Loneliness Scale (RomLon scale).

**Method:**

The current research was conducted in a series of three consecutive phases involving 854 participants (M_age_ = 26.25 years, SD = 8.160; women = 64.9%). The validation of the RomLon scale involved exploratory and confirmatory factor analyses and Rasch model analysis for item‒response theory, along with convergent, divergent, and predictive validity.

**Results:**

The RomLon scale, comprising four items (English) in a single factor, demonstrated excellent reliability (α = 0.919; ICC = 0.963). The model fit indices, such as CFI (0.997), TLI (0.992), RMSEA (0.065), and SRMSR (0.009), showed strong validity. Convergent validity and divergent validity were demonstrated by the strong correlation (*p* < 0.001) of the scale with the UCLA Loneliness Scale (*r* = 0.383) and the psychosocial life satisfaction scale (*r* = -0.318), respectively. Romantic loneliness predicted hopelessness and sexual distress, was positively correlated with age and education, and was greater in men and married individuals than in women and unmarried individuals.

**Conclusion:**

The RomLon scale fills a critical gap in psychological assessment by providing a reliable tool to measure romantic loneliness, which is distinct from general loneliness scales. Its strong psychometric properties make it valuable for research and clinical applications across diverse cultures, with implications for psychosocial health, life satisfaction, and relational wellbeing.

## Introduction

The evolving landscape of modern relationships, shaped by the rise of online dating platforms and the prevalence of hookup culture, has significantly altered traditional romantic experiences [1] and common mate-attraction strategies [2, 3]. While digital technology has expanded opportunities for romantic connections through platforms like Tinder, Bumble, and Grindr [4], it has also introduced new psychological challenges [5], including romantic loneliness. Many individuals navigating online dating and casual encounters experience emotional dissatisfaction, unfulfilled romantic expectations, a sense of detachment, depression, anxiety, and fear of being single, contributing to feelings of loneliness despite increased connectivity [6–8]. Moreover, behaviors such as ghosting and breadcrumbing, which are prevalent in contemporary dating culture [9], often exacerbate these adverse emotional experiences, leaving individuals feeling disconnected and emotionally unfulfilled. Poor dating performance is also common among men and women which significantly predicts various forms of singlehood, especially among younger individuals from various cultures [10]. Beyond the sphere of dating and casual relationships, romantic loneliness is also a growing concern within marital relationships [11]. While marriage is traditionally viewed as a stable source of emotional intimacy and companionship, many individuals experience a lack of romantic fulfillment, leading to profound emotional distress [12]. The absence of romance in long-term relationships can manifest as emotional detachment, unmet emotional needs, and a sense of relational emptiness, ultimately affecting mental well-being and relationship satisfaction.

Romance can be defined in various ways depending on cultural, social, and individual perspectives [13]. Generally, romance refers to a deep emotional connection or attachment between individuals characterized by feelings of love, affection, and intimacy [14]. It involves expressions of admiration, desire, and attraction toward another person, often accompanied by gestures of affection, thoughtfulness, and devotion [15]. Romance typically involves strong feelings of love, affection, and emotional connection between partners [16]. It goes beyond mere friendship or companionship and often involves a deep emotional bond [14]. Romantic aspects also play a vital role in marital satisfaction, well-being, and high self-esteem [17, 18]. Romance is often marked by a sense of intimacy, both emotional and physical. This includes sharing personal thoughts and feelings, as well as engaging in physical expressions of affection such as intimate touch, kissing, hugging, and cuddling [15, 19]. Romance is fueled by attraction and desire toward the other person. This can include physical attraction and admiration for one’s personality, character, and qualities [20]. Romantic relationships often involve thoughtful gestures and acts of kindness aimed at expressing love and appreciation for the partner [15, 20]. These gestures can range from simple compliments and surprises to grand romantic gestures [21]. Romance is often associated with a sense of commitment and devotion to the relationship [14]. This may involve making sacrifices, prioritizing the well-being of the partner, and working together to overcome challenges [21]. Romantic relationships are built on shared experiences and meaningful moments that create lasting memories [15, 20, 21]. These shared experiences help strengthen the bonds between partners and deepen their connections over time. Importantly, the experience and expression of romance can vary widely among individuals and cultures [22]. What one person finds romantic may not resonate with another, and different cultural norms and values can influence how romance is perceived and expressed. However, at its core, romance is about fostering deep emotional connections and nurturing loving relationships between individuals [14].

Loneliness is a significant public health issue [23] that is closely linked to adverse physical and mental health outcomes. It can be categorized into emotional loneliness: missing an intimate relationship; social loneliness: missing a broader social network; and existential loneliness: the realization of human isolation [24]. Loneliness has been associated with negative mental health outcomes, particularly among young adults [25, 26], including depression [27] and feelings of isolation [28]. In the existing body of literature, there is a significant gap in the measurement of romantic loneliness. While general loneliness has been widely studied and some existing measures address adjacent constructs, scales specifically designed to capture the emotional and psychological nuances of romantic loneliness remain scarce. Loneliness, as a broad construct, has been extensively studied, with numerous tools being widely utilized across diverse populations. These scales, however, predominantly focus on loneliness in broader social contexts, without delving into the specific experience of loneliness within the framework of romantic relationships.

Romantic loneliness refers to the distressing feeling of lacking a meaningful romantic relationship despite a desire for intimacy and connection [29]. It differs from general loneliness, as it specifically stems from unmet emotional and physical needs in a romantic context [30]. It involves not only the absence of a partner but also a lack of emotional intimacy, affection, and companionship, which are crucial aspects of romantic bonding. Research has shown that romantic loneliness is a unique subdimension of loneliness with significant implications for emotional well-being and mental health [24, 31]. Individuals experiencing romantic loneliness are more likely to report symptoms of depression, anxiety, reduced life satisfaction, and suicidal ideation [32, 33]. The lack of romantic attachment undermines core psychological needs such as belongingness, validation, and love [34]. Moreover, romantic loneliness may contribute to negative self-perceptions, including feelings of undesirability, low self-worth, and emotional vulnerability [35]. These psychosocial consequences are compounded in societies where romantic relationships are idealized as markers of success and identity, further exacerbating the distress of individuals who remain single or feel romantically unfulfilled [36]. Despite its significance, romantic loneliness has not been systematically addressed in many existing loneliness scales. The absence of a scale dedicated to romantic loneliness is particularly concerning, as it neglects the psychological impact of relational distress, which is distinct from general social or familial isolation. This gap in measurement tools hinders a deeper understanding of the specific factors contributing to romantic loneliness, as well as the identification of individuals who may be vulnerable to this form of isolation. Given the growing body of evidence that links romantic loneliness with negative psychological outcomes, the need for a valid, reliable, and specialized instrument to assess romantic loneliness has become more urgent. To address this gap, the aim of the present study was to develop and validate a concise instrument specifically designed to measure romantic loneliness.

## Method

The Romantic Loneliness Scale (RomLon scale) was developed and validated through three distinct phases in the present study. The first phase focused on the initial development of the scale, followed by exploratory factor analysis (EFA). The second phase involved conducting a confirmatory factor analysis (CFA) and establishing its divergent validity. The final phase assessed the convergent and predictive validity of the RomLon scale to further establish its reliability and applicability.

### Development of the RomLon scale

An initial item pool of six items was constructed for the RomLon scale (Table 1). These items were developed in accordance with the earlier literature to measure romantic desires (I easily become captivated by romantic thoughts), feelings of sadness (I feel sad when I think about being romantically alone), exclusion and comparison (I feel left out when I see other couples expressing affection), envy and inadequacy (I feel like others are having better romantic experiences than I am), anxiety and fear (I worry that I’ll never find romance in my life), and sense of emptiness (I feel like something is missing from my life because I don’t have romance) related to romantic loneliness [29, 34, 37–52]. The decision to maintain the brevity of the scale was driven by several key considerations, particularly cultural sensitivity surrounding the expression of romantic desires [53, 54]. In many cultures, discussing romantic emotions or desires, especially in the context of intimate or sexual relationships, can be viewed as taboo or uncomfortable [55]. This cultural restraint often leads to reluctance in openly addressing emotional and psychological aspects of romantic relationships, making it crucial to design an instrument that would be perceived as respectful and non-invasive [56]. Given that romantic loneliness specifically pertains to feelings of isolation or emotional disconnection within romantic relationships [29, 30], it was deemed essential to avoid including items that might prompt responses related to sexual or instinctual desires, which could inadvertently introduce discomfort or cause respondents to disengage. By focusing solely on emotional and relational aspects of romantic loneliness, the scale ensures that it remains culturally sensitive and avoids delving into potentially sensitive or stigmatized areas, such as sexual intimacy or personal attraction. This approach also minimizes the risk of introducing bias or social desirability effects, where participants might underreport or distort their responses due to fear of judgment. Additionally, a brief scale enables quicker completion, making it more accessible and less burdensome for respondents while still capturing the essential dimensions of romantic loneliness.

**Table 1:** Development of the RomLon scale

No.	Item	Theme	Importance/Significance to RomLon scale	Final version
1	I easily become captivated by romantic thoughts.	Romantic Desires	Assesses the intensity of longing for romance and emotional engagement with romantic thoughts.	Item retained
2	I feel sad when I think about being romantically alone.	Feelings of Sadness	Directly addresses the emotional impact of romantic loneliness and its effects on well-being.	Item retained
3	I feel left out when I see other couples expressing affection.	Exclusion and Comparison	Highlights feelings of isolation and the impact of social comparisons on self-esteem.	Item retained
4	I feel like others are having better romantic experiences than I am.	Envy and Inadequacy	Reflects feelings of inadequacy and the perception of others’ more fulfilling romantic lives.	Item retained
5	I worry that I’ll never find romance in my life.	Anxiety and Fear	Focuses on fears regarding future romantic prospects, which can contribute to overall distress.	Item discarded
6	I feel like something is missing from my life because I don’t have romance.	Sense of Emptiness	Captures the overall sense of lack or void in life due to the absence of romantic relationships.	Item discarded

**Notes**: References for the literature that helped in developing these items = [29, 34, 37–52]

The initial items were presented to a panel of three expert psychologists to determine the facial validity of the RomLon scale. These experts had sufficient experience in psychosocial and psychometric studies, including extensive backgrounds in scale development, clinical assessment, and relational psychology. Each expert held doctoral qualifications, had published peer-reviewed research on psychological measurement and interpersonal constructs, and possessed over a decade of experience in evaluating the psychometric soundness of psychological instruments. Their combined expertise ensured a rigorous evaluation of item clarity, conceptual relevance, and contextual appropriateness for assessing romantic loneliness. The panel confirmed that the items were valid for measuring romantic loneliness. Their agreement was also measured by interrater reliability, which reflected substantial agreement between the raters (Cohen’s weighted kappa = 0.714; Fleiss’s kappa = 0.723; Krippendorff’s alpha = 0.731). Two items (Table 1) were discarded during the exploratory factor analysis because they did not have the required thresholds for validity: communalities less than 0.4 or cross loadings between factors above 0.2 [57]. The finalized RomLon scale comprises four items comprising a single factor that was further validated through confirmatory factor analysis along with establishing the convergent, divergent, and predictive validity of the RomLon scale.

### Participants

The present study was conducted in three consecutive phases. A total of 854 adults from Rawalpindi and Islamabad, Pakistan, participated in the study. The sample comprised 300 men (35.1%) and 554 women (64.9%). The participants’ ages ranged from 18 to 71 years, with a mean age of 26.25 years (SD = 8.160). In terms of marital status, 637 (74.6%) were unmarried, whereas 55 (18.4%) were married. Educational qualifications varied from five years of formal schooling to a doctoral degree, with the average level of education being graduation (14 years of formal education).

The first phase of the study involved 311 participants (men = 81, 26%; women = 230, 74%; unmarried = 239, 76.8%; married = 72, 23.2%; age = 18–61 years, M = 25.27, SD = 6.73; education = middle to doctorate, M = graduation). The second phase involved 244 participants (men = 110, 45.1%; women = 134, 54.9%; unmarried = 154, 63.1%; married = 90, 36.9%; age = 18–71 years, M = 31.377, SD = 9.851; education = primary to doctorate, M = graduation). The final phase involved 299 participants (men = 109, 36.5%; women = 190, 63.5%; unmarried = 244, 81.6%; married = 55, 18.4%; age = 18–60 years, M = 23.10, SD = 5.63; education = middle to doctorate, M = graduation).

A convenience sampling technique was employed to recruit participants for all the phases. The researchers individually approached participants during visits to various academic institutions, as well as government and private offices. Participation in the study was entirely voluntary, and informed consent was obtained from all participants before their involvement. The inclusion criteria required individuals to be (a) at least 18 years old and (b) proficient in responding to questionnaires in English. The study employed convenience sampling, with participants individually approached in academic institutions, government offices, and private organizations. Participation was voluntary, with informed consent obtained beforehand. While selection bias is a concern in convenience sampling, efforts were made to ensure inclusive representation across diverse age groups. The nonsystematic recruitment process minimized favoritism, and voluntary participation allowed individuals to exercise personal agency, reducing the risk of systematic exclusion.

### Sample size calculations

The sample size for this research was determined on the basis of established guidelines for EFA and CFA and to establish predictive validity. For EFA, a minimum of five participants per item is recommended [58, 59]. Given the initial pool of six items, the minimum sample size required for EFA was calculated to be 30 participants. Our data collection exceeded this requirement by involving 311 participants for EFA. For CFA, the sample size requirement is generally greater, with the guideline recommending at least 10 participants per estimated parameter in the model [60]. The CFA model in this study included four items and a single factor, resulting in eight parameters to be estimated (4 factor loadings and 4 error variances). Thus, the minimum sample size required for CFA was 80 participants. This requirement was surpassed, with the second phase involving 244 participants, ensuring sufficient power to validate the factor structure. Convergent, divergent, and predictive validity is typically assessed through correlation analyses and regression, which necessitate adequate statistical power to detect meaningful relationships between the scale scores and relevant outcome variables. A moderate effect size (f² = 0.15) in a multiple regression analysis with three to five predictor variables requires a minimum sample of approximately 77 to 92 participants to achieve 80% power at an alpha level of 0.05 [61]. However, to increase the robustness and generalizability of the findings, researchers often target a larger sample. The data collected in the second and third phases of the current study adhered to this principle, with 244 and 299 participants, respectively.

### Instruments

#### UCLA loneliness scale (version 3)

Loneliness is a broad psychological state characterized by a perceived discrepancy between desired and actual social relationships [62]. Romantic loneliness, as a specific subtype, pertains to the absence or insufficiency of meaningful romantic connections, leading to feelings of emotional isolation within the romantic domain [29]. The UCLA Loneliness Scale, version 3 [63], was utilized to establish the convergent validity of the RomLon scale. The UCLA loneliness scale (version 3) is a widely used self-report measure designed to assess subjective feelings of loneliness and social isolation. It consists of 20 items rated on a four-point Likert scale, capturing both the frequency and intensity of experience. The scale has strong psychometric properties, with high internal consistency (Cronbach’s alpha typically exceeding 0.90) and test-retest reliability, indicating its stability over time. The scale also exhibited excellent reliability in the current research (Cronbach’s alpha = 0.915).

#### Psychosocial life satisfaction scale

Romantic loneliness and life satisfaction are theoretically opposing constructs, as the presence of meaningful romantic connections is often linked to overall wellbeing and fulfillment [64]. The psychosocial life satisfaction scale [65] was used to measure the divergent validity of the RomLon scale. The psychosocial life satisfaction scale comprises 5 items and uses a 7-point Likert scale for responses (extremely unsatisfied to extremely satisfied). The developer of the scale claimed the scale to be highly reliable (Cronbach’s alpha = 0.872; item‒total correlations were reported to be highly significant = *p* < 0.001) and valid (CFI = 0.991; TLI = 0.981; RMSEA = 0.066). The scale also exhibited excellent reliability in the current research (Cronbach’s alpha = 0.923).

#### Hopelessness scale

Romantic loneliness may serve as a significant predictor of hopelessness, as unfulfilled romantic needs can lead to persistent feelings of emotional emptiness, social disconnection, and diminished optimism about future relationships [66]. The predictive validity of the RomLon scale was established by correlating it with the hopelessness scale. The hopelessness scale [67, 68] is a popular tool for measuring negative expectations about the future. The scale has been regarded as a critical measure in both research and clinical settings. The 20 true-false items examine three different aspects of hopelessness, including feelings about the future, loss of motivation, and expectations. Each question is given a score of 0 or 1, with higher overall scores signifying hopelessness. Researchers have regarded the scale as highly reliable and valid [67, 68]. Most factor analyses corroborate the scale’s three-component structure; however, some show a single dominant hopelessness factor [69]. The scale is positively correlated with depression and negatively correlated with hope and optimism. The scale is applicable to many populations because it has been developed and verified in several cultures. The scale also exhibited satisfactory reliability in the current research (Cronbach’s alpha = 0.762).

#### Sexual distress scale

Romantic loneliness may predict sexual distress by fostering feelings of emotional isolation, unmet intimacy needs, and dissatisfaction with one’s romantic and sexual experiences [70]. The sexual distress scale [71] was used to measure the predictive validity of the RomLon scale. The sexual distress scale comprises eight items constituting a single factor. It uses a 5-point Likert scale for responses (never to always). The developer of the scale claimed the scale to be highly reliable (Cronbach’s alpha = 0.907; item‒scale and item‒total correlations were reported to be highly significant = *p* < 0.001) and valid (CFI = 0.913). The scale also exhibited excellent reliability in the current research (Cronbach’s alpha = 0.936).

### Ethical considerations

This research was conducted in accordance with established ethical guidelines (as outlined in the 1964 Helsinki Declaration and its subsequent amendments) to ensure the rights, dignity, and wellbeing of all participants. Ethical approval was obtained from the departmental review committee at COMSATS University (Code: CUI-ISB/HUM/ERC-CPA/2024-20) before data collection. Participation in the study was entirely voluntary, and informed consent was obtained from all participants prior to their inclusion. The participants were briefed about the purpose of the research, the confidentiality of their responses, and their right to withdraw at any stage without any consequences. Anonymity was maintained, and the participants were not asked about any personal information. No deceptive practices were used, and the participants did not face any potential risks or harm. The collected data were securely stored and used solely for research purposes, ensuring privacy and confidentiality.

### Analysis

The data collected were recorded and analyzed via the Statistical Package for Social Sciences (SPSS-26) and R for statistical computing (R version 4.3.2). A rigorous data cleaning process was undertaken. There were no missing values in the dataset. All participants provided complete responses on the RomLon scale and associated variables. The values of skewness and kurtosis for all items were found to be within the acceptable range, indicating that the data did not significantly deviate from normality.

To assess the reliability and validity of the RomLon scale, both EFA and CFA were conducted. In the EFA, maximum likelihood was employed with no rotation, with the number of factors determined on the basis of the eigenvalues. EFA was used to examine the factor structure, extraction values, Bartlett’s test of sphericity (BTS), Kaiser‒Meyer‒Olkin (KMO) measure of sampling adequacy, CFI, TLI, RMSEA, SRMR, and total variance explained.

For the CFA, the maximum-likelihood extraction technique was utilized without rotation. Model fit was assessed via various fit indices, including the chi-square test, CFI, TLI, Bentler-Bonett nonnormed fit index (NNFI), Bentler-Bonett normed fit index (NFI), Parsimony normed fit index (PNFI), Bollen’s relative fit index (RFI), Bollen’s incremental fit index (IFI), the relative noncentrality index (RNI), RMSEA, SRMR, the goodness-of-fit index (GFI), McDonald’s fit index (MFI), and the expected cross-validation index (ECVI). Additionally, the KMO test, Bartlett’s test of sphericity, the heterotrait–monotrait (HTMT) ratio, Cronbach’s alpha, and McDonald’s omega were computed to evaluate the scale’s reliability and construct validity.

To assess the correlation between variables, Pearson’s correlation coefficient was employed. Simple regression analysis was conducted to examine the predictive validity of the RomLon scale. Independent samples t tests were performed to compare the levels of romantic loneliness based on gender and marital status. Rasch model analysis for item response theory (IRT) was employed to evaluate the psychometric properties and item fit of the RomLon scale.

## Results

### Reliability

The RomLon scale demonstrated excellent internal consistency across both exploratory and confirmatory phases. In the Exploratory Factor Analysis (EFA), the scale yielded a Cronbach’s alpha of 0.846 (Table 2), indicating a high level of reliability. This reliability further improved in the Confirmatory Factor Analysis (CFA), where the Cronbach’s alpha reached 0.919 (Table 2). The enhanced internal consistency observed in the CFA phase can be attributed to the refinement process, during which two items that demonstrated suboptimal psychometric properties were excluded based on EFA results. The four most robust and validated items were retained for the CFA which resulted in strengthening the overall coherence and reliability of the scale. The item-total correlations (ranging from 0.791 to 0.860 with *p* < 0.001; mean = 0.827) of the RomLon scale items, as measured via EFA, demonstrated a high degree of internal consistency (*p* < 0.001). The test-retest reliability of the RomLon scale after a two-week interval with the same participants (*n* = 30) was excellent (interclass correlation type = ICC3,1; point estimate = 0.963; 95% CI = 0.923—0.982).

**Table 2:** Descriptive statistics, reliability, and data accuracy (*n* = 854)

Variable	Items	α	M	SD	%	Range		Skewness	Kurtosis
						Potential	Actual		
*Phase 1*									
RomLon-Scale	4	0.846	18.356	6.034	65.557	4–28	4–28	-0.481	-1.068
*Phase 2*									
RomLon-Scale	4	0.919	21.163	5.506	75.582	4–28	4–28	-1.356	0.966
Psychosocial Life Satisfaction Scale	5	0.923	22.286	7.917	63.674	5–35	6–35	0.051	-1.209
*Phase 3*									
RomLon-Scale	4	0.768	14.244	5.996	50.871	4–28	4–28	0.187	-0.774
Sexual Distress Scale	8	0.936	13.619	7.313	34.047	8–40	8–40	1.447	1.187
Hopelessness Scale	20	0.762	6.442	4.054	32.208	0–20	0–19	0.514	-0.265
UCLA Loneliness Scale	20	0.915	26.070	12.881	43.450	0–60	0–59	0.064	-0.445

**Notes**: *n* = Number of participants; α = Cronbach’s alpha; M = Mean; SD = Standard deviation

Phase 1: *n* = 311; men = 81, 26%; women = 230, 74%; unmarried = 239, 76.8%; married = 72, 23.2%; age = 18–61 years, M = 25.27, SD = 6.73; education = middle to doctorate, M = graduation

Phase 2: *n* = 244; men = 110, 45.1%; women = 134, 54.9%; unmarried = 154, 63.1%; married = 90, 36.9%; age = 18–71 years, M = 31.377, SD = 9.851; education = primary to doctorate, M = graduation

Phase 3: *n* = 299; men = 109, 36.5%; women = 190, 63.5%; unmarried = 244, 81.6%; married = 55, 18.4%; age = 18–60 years, M = 23.10, SD = 5.63; education = middle to doctorate, M = graduation

Total phases: *n* = 854; men = 300, 35.1%; women = 554, 64.9%; unmarried = 637, 74.6%; married = 217, 25.4%; age = 18–71 years, M = 26.25, SD = 8.160; education = primary to doctorate, M = graduation

### Exploratory factor analysis (EFA)

In the EFA (Table 3), Promax rotation was employed, with the number of factors determined on the basis of the eigenvalues. The sampling adequacy was notable (Table 3; *n* = 311; KMO = 0.818 for the overall scale, which ranged from 0.784 to 0.855 for individual items). The adequacy of correlations between items was highly significant (Table 3; BTS: χ² = 500.933, *df* = 6, *p* < 0.001). Two items were discarded during the exploratory factor analysis because they did not have the required thresholds for validity: communalities less than 0.4 or cross loadings between factors above 0.2 [57]. The final RomLon scale comprises four items constituting a single factor. The factor loading of these items ranged from 0.695 to 0.830 (Table 3).

**Table 3:** Exploratory factor analysis and item‒total correlations (Phase 1; *n* = 311)

Item No.	Item	Factor Structure	Extraction	Item-total Correlation
1	I easily become captivated by romantic thoughts.	0.830	0.688	0.860^***^
2	I feel sad when I think about being romantically alone.	0.778	0.605	0.836^***^
3	I feel left out when I see other couples expressing affection.	0.743	0.552	0.823^***^
4	I feel like others are having better romantic experiences than I am.	0.695	0.484	0.791^***^

**Notes**: *** *p* < 0.001

Extraction: Maximum likelihood with no rotation

Bartlett’s Test of Sphericity: χ² = 500.933, df = 6, *p* < 0.001 Kaiser-Meyer-Olkin Measure of Sampling Adequacy = 0.818 (ranged from 0.784 to 0.855 for individual items) Chi-square test: value = 0.245; df = 2; *p* = 0.885

Total variance explained: 58.233 Response sheet: extremely false (scored 1), false (scored 2), slightly false (scored 3), not sure (scored 4), slightly true (scored 5), true (scored 6), extremely true (scored 7)

### Confirmatory factor analysis (CFA)

**Fig. 1:**
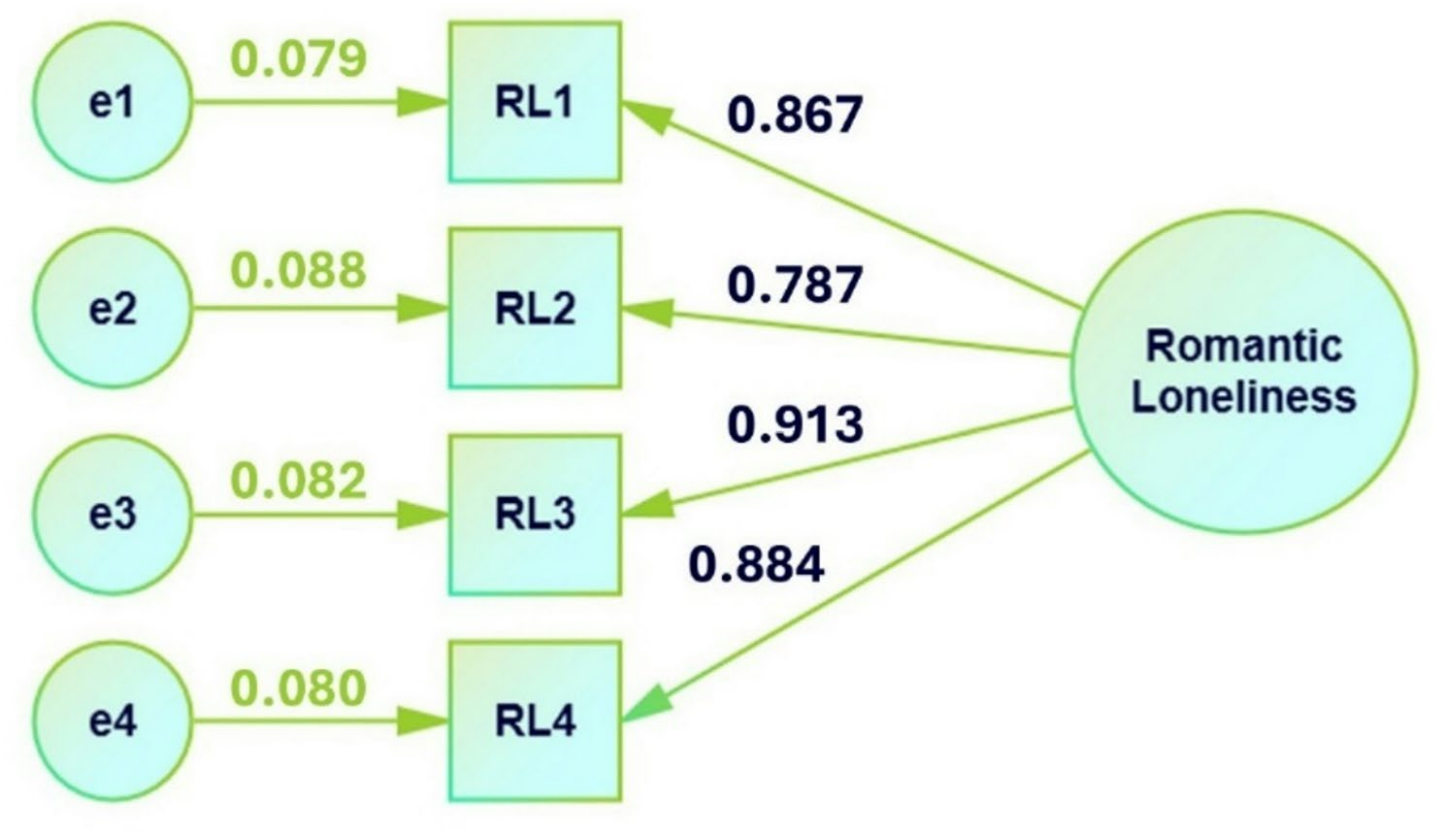
Confirmatory factor analysis (Phase 2; *n* = 244)

CFA was conducted on four items to test a single-factor model (Fig. 1). The maximum-likelihood extraction technique was utilized without rotation. To assess normality of data, we evaluated univariate skewness (values for the four items = -1.382, -1.240, -1.190, and − 0.934) and kurtosis (values for the four items = 1.352, 0.829, 0.812, and 0.097) for the RomLon Scale to assess the distributional properties of individual items. This approach is aligned with standard practices in psychometric analysis when the primary concern is to ensure that each variable does not deviate significantly from normality. Skewness values less than ± 3 and kurtosis values less than ± 10 are generally considered acceptable for maximum likelihood estimation in structural equation modeling [60]. The sampling adequacy was also notable (Table 4; *n* = 244; KMO = 0.849 for the overall scale, which ranged from 0.812 to 0.903 for individual items). The adequacy of correlations between items was highly significant (Table 4; BTS: χ² = 802.631, *df* = 6, *p* < 0.001). The factor loadings were statistically significant (*p* < 0.001) and ranged from 0.787 to 0.913 (Table 4), indicating that the items were strongly related to the underlying factor. The average variance extracted was 0.746, which demonstrated adequate convergence. The reliability was also excellent (coefficient ω = 0.921; coefficient α = 0.920). The CFA model demonstrated good fit according to several fit indices, such as CFI (0.997), TLI (0.992), NNFI (0.992), NFI (0.995), PNFI (0.332), RFI (0.984), IFI (0.984), RNI (0.997), RMSEA (0.065), SRMR (0.009), GFI (0.999), and MFI (0.996).

**Table 4:** Confirmatory factor analysis (Phase 2; *n* = 244)

Factor	Item	Factor loadings					Residual variances								
		Estimate	SE	z	*p*		Estimate		SE		z		*p*		
F1	RL1	0.867	0.079	17.523	< 0.001			0.248		0.072		8.652		< 0.001	
	RL2	0.787	0.088	15.084	< 0.001			0.381		0.109		10.050		< 0.001	
	RL3	0.913	0.082	19.076	< 0.001			0.167		0.070		6.949		< 0.001	
	RL4	0.884	0.080	18.114	< 0.001		0.218		0.070		8.266		< 0.001		

**Notes**: Extraction was performed using the Maximum-likelihood extraction technique with no rotation

Chi-square test: Baseline model: χ² = 812.228, *df* = 6, Factor model: χ² = 4.257, *df* = 2, *p* > 0.05

Additional Fit Measures: Comparative Fit Index (CFI): 0.997; Tucker-Lewis Index (TLI): 0.992; Bentler-Bonett Non-normed Fit Index (NNFI): 0.992; Bentler-Bonett Normed Fit Index (NFI): 0.995; Parsimony Normed Fit Index (PNFI): 0.332; Bollen’s Relative Fit Index (RFI): 0.984; Bollen’s Incremental Fit Index (IFI): 0.997; Relative Noncentrality Index (RNI): 0.997; Information Criteria: Log-likelihood: -1656.732, Number of free parameters: 12, Akaike (AIC): 3337.464, Bayesian (BIC): 3380.556, Sample-size adjusted Bayesian (SSABIC): 3342.509; Root mean square error of approximation (RMSEA): 0.065; RMSEA 90% CI lower bound: 0.000; RMSEA 90% CI upper bound: 0.152; RMSEA p-value: 0.288; Standardized root mean square residual (SRMR): 0.009; Hoelter’s critical N (α = 0.05): 378.195; Hoelter’s critical N (α = 0.01): 580.841; Goodness of fit index (GFI): 0.999; McDonald fit index (MFI): 0.996; Expected cross-validation index (ECVI): 0.105; Kaiser-Meyer-Olkin (KMO) Test: Overall KMO: 0.849 (ranged from 0.812 to 0.903 for individual items); Bartlett’s Test of Sphericity: χ² = 802.631, *df* = 6, *p* < 0.001; R-Squared: Explained variance (R²) for the four items ranged from 0.619 to 0.833; Heterotrait-monotrait ratio = 1; Total variance extracted = 0.746; Coefficient α = 0.920; Coefficient ω = 0.921

### Convergent, divergent, and predictive validity

Convergent validity was demonstrated by the correlation of the RomLon scale with the UCLA loneliness scale (Table 5; *r* = 0.383, *p* < 0.001). Divergent validity was established through an inverse correlation between the RomLon scale and psychosocial life satisfaction scale (Table 5; *r* = -0.318, *p* < 0.001). The predictive validity of the RomLon scale was established through its predictive values for hopelessness (Table 6; *β =* 0.285; *p* < 0.001) and sexual distress (Table 6; *β =* 0.466; *p* < 0.001).

**Table 5:** Correlations

	RomLon-Scale
Sexual Distress Scale	0.466^***^
Hopelessness Scale	0.285^***^
UCLA Loneliness Scale	0.383^***^
Psychosocial Life Satisfaction Scale	-0.318^***^
Age	0.228^***^
Education	0.072^***^

**Notes**: *** *p* < 0.001

**Table 6:** Romantic loneliness as a predictor of hopelessness and sexual distress (simple regression)

	*R*	*R* ^2^	Adj. *R*^2^	df	F	B	SE B	β	t	*p*
Hopelessness	0.285	0.081	0.078	297	26.295	0.194	0.038	0.285	5.128	< 0.001
Sexual distress	0.466	0.217	0.214	297	82.252	0.571	0.063	0.466	9.069	< 0.001

**Notes**: *R* = Multiple correlation coefficient; *R*^*2*^ = Coefficient of determination; *Adj.* = Adjusted; *df* = Degrees of freedom; *F* = F statistic; *B =* Unstandardized regression coefficient; *SE =* Standard error; *β =* Standardized regression coefficient; *t =* t statistic; *p =* p value

### Rasch model analysis for item response theory (IRT)

Rasch model analysis for item response theory (IRT) was employed to evaluate the psychometric properties and item fit of the RomLon scale (Table 7). The item mean scores ranged from 4.22 to 4.61, indicating a moderate level of agreement with the items. The item measure values, which represent the item difficulty estimates, ranged from − 2.63 to 4.01 logits. The infit and outfit mean‒square statistics, which assess the degree to which the observed data conform to the expected model, were within the acceptable range of 0.6–1.4 for all items [72–74], suggesting adequate item fit. The point-biserial correlations, which indicate the extent to which an item discriminates between individuals with high and low levels of the measured trait, ranged from 0.787 to 0.832, indicating good item discrimination. The threshold parameters represent the points on the latent trait continuum where the likelihood of responding in one category is equal to the likelihood of responding in the adjacent category. The threshold values for all the items monotonically increased, indicating that the rating scale categories were ordered appropriately. The person separation reliability, an indicator of the ability of the scale to distinguish between individuals with different levels of the measured trait, was 0.770, which is considered acceptable for group-level comparisons [72–74].

**Table 7:** Item response analysis of the PLSS using entire samples from the four studies (*n* = 1297)

Item statistics – RSM							Thresholds delta						
Item	Item mean	Measure	S.E. Measure	Infit	Outfit	Point biserial	1	2	3	4	5	6	7
RL1	4.61	-0.1595	0.0326	1.000	0.946	0.807	-2.63	-0.771	-1.07	-1.22	-0.142	1.82	4.01
RL2	4.37	0.0583	0.0324	0.768	0.740	0.832	-2.63	-0.771	-1.07	-1.22	-0.142	1.82	4.01
RL3	4.22	0.1906	0.0324	0.977	0.905	0.807	-2.63	-0.771	-1.07	-1.22	-0.142	1.82	4.01
RL4	4.53	-0.0894	0.0326	1.089	1.028	0.787	-2.63	-0.771	-1.07	-1.22	-0.142	1.82	4.01

**Notes**: Rasch model analysis for item response theory. Model information – RSM: AIC: 8717; BIC: 8867; CAIC: 8899; Log likelihood: -4326; Parameters: 32; Persons: 819; Person separation reliability using the eRm R package: SSD: 1.16; MSE: 0.266; Reliability: 0.770

### Romantic loneliness, age, education, gender, and marital status

In addition to developing and validating the RomLon scale, the current study also provides additional findings on the linkages between romantic loneliness, age, education, gender, and marital status. The study revealed a significant positive correlation between romantic loneliness and age (Table 5; *r* = 0.228; *p* < 0.001), indicating that older individuals tend to experience higher levels of romantic loneliness. The study also revealed a significant positive correlation between romantic loneliness and education (Table 5; *r* = 0.072; *p* < 0.001), indicating that highly educated individuals tend to experience higher levels of romantic loneliness. Compared with women, men presented significantly greater levels of romantic loneliness, with moderate effect (Table 8; M = 19.360, SD = 6.060, % = 69.142 vs. M = 16.830, SD = 6.542, % = 60.107; *p* < 0.001; Cohen’s d = 0.397). Compared with their unmarried counterparts, married individuals presented significantly greater levels of romantic loneliness, with moderate effect (Table 9; M = 19.235, SD = 6.051, % = 68.696 vs. M = 17.203, SD = 6.554, % = 61.439; *p* < 0.001; Cohen’s d = 0.316).

**Table 8:** Differences in romantic loneliness by gender (*n* = 854)

Variables	Men (*n* = 300)				Women (*n* = 554)				t(852)	*p*	Cohen’s d
	M	SD	%		M	SD	%				
Romantic Loneliness	19.360	6.060	69.142		16.830	6.542	60.107		5.533	0.000	0.397

**Table 9:** Differences in romantic loneliness by marital status (*n* = 854)

Variables	Unmarried (*n* = 637)				Married (*n* = 217)				t(852)	*p*	Cohen’s d
	M	SD	%		M	SD	%				
Romantic Loneliness	17.203	6.554	61.439		19.235	6.051	68.696		4.021	0.000	0.316

## Discussion

Although related constructs have been explored in prior research, including general loneliness and the fear of being single, there remains a lack of dedicated measures that directly assess romantic loneliness as a unique form of emotional distress stemming from unmet needs for romantic intimacy and connection [29, 30]. One apparently similar scale is the Fear of Being Single Scale - FOBS [75]. The FOBS is a prominent measure in the domain of romantic relationship research. However, the construct assessed by the RomLon scale is conceptually and empirically distinct. FOBS primarily captures anxiety about remaining single in the future and is framed around relationship status insecurity, particularly for single individuals, although adaptations have been developed for those in relationships [75]. In contrast, the RomLon scale is designed to assess romantic loneliness, which refers to the emotional distress, cognitive preoccupation, and perceived social exclusion experienced by individuals who feel unfulfilled in their romantic lives, regardless of their current relationship status. The RomLon scale integrates theoretical insights from loneliness research, attachment theory, and social comparison frameworks, and its items capture multidimensional aspects of romantic longing and dissatisfaction. While FOBS reflects anticipatory fear about singlehood, RomLon emphasizes lived emotional experience, making it a broader and more affectively grounded construct. Furthermore, RomLon demonstrated strong psychometric properties, including high reliability, validity, and predictive power for indicators such as hopelessness and sexual distress.

The RomLon scale was developed and validated in the present study through a series of three consecutive phases. The scale underwent rigorous reliability and validity testing through both EFA and CFA. It has high internal consistency and test‒retest reliability. Through statistical analyses, the final version of the RomLon scale was refined to include four items constituted under a single factor. It comprises four items (in English) constituting a single factor. The scale was administered using a seven-point Likert format (extremely false to extremely true). The RomLon scale demonstrated high reliability in both the EFA and the CFA (Cronbach’s alpha: 0.846 & 0.919). The item–total correlations (ranging from 0.791 to 0.860 with *p* < 0.001; mean = 0.827) of the RomLon scale items demonstrated a high degree of internal consistency during the EFA. The test-retest reliability of the RomLon scale after a two-week interval with the same participants (*n* = 30) was excellent (interclass correlation type = ICC3,1; point estimate = 0.963; 95% CI = 0.923–0.982). In the CFA, several model fit indices, such as the comparative fit index (CFI = 0.997), Tucker‒Lewis index (TLI = 0.992), root mean square error of approximation (RMSEA = 0.065), and standardized root mean square residual (SRMR = 0.009), showed strong validity. Strong convergent validity was demonstrated by the strong correlation of the scale with the UCLA loneliness scale (*r* = 0.383, *p* < 0.001). Divergent validity was established through a strong inverse correlation between the RomLon scale score and psychosocial life satisfaction scale score (*r* = -0.318, *p* < 0.001). The predictive validity of the RomLon scale was established through its strong predictive values for hopelessness (*β =* 0.285; *p* < 0.001) and sexual distress (*β =* 0.466; *p* < 0.001).

The four items of the RomLon scale are fully capable of capturing the key dimensions of romantic loneliness by assessing an individual’s emotional responses, cognitive patterns, and social comparisons related to romantic relationships. Each item reflects a distinct yet interconnected aspect of romantic longing and dissatisfaction, making the scale a valid measure of romantic loneliness and readiness. The first item (I easily become captivated by romantic thoughts) measures heightened romantic sensitivity and cognitive preoccupation with romance, indicating an underlying desire for intimacy. Individuals who frequently engage in romantic thoughts may experience a strong unmet need for emotional connection, reflecting their loneliness and readiness for romantic engagement [29]. Studies on attachment and romantic longing highlight that individuals with increased preoccupation with romantic relationships may experience greater emotional distress when their need for closeness is unmet [34, 45, 46]. Furthermore, research on relationship readiness suggests that individuals who frequently think about romance may be psychologically primed for romantic engagement, as such cognitions indicate an openness to forming deep emotional bonds [47, 48]. The second item (I feel sad when I think about being romantically alone) captures the emotional distress associated with a lack of romantic companionship. Feelings of sadness in response to romantic solitude highlight an individual’s recognition of an unfulfilled need, which is central to romantic loneliness. Research on loneliness and emotional wellbeing suggests that unfulfilled desires for intimacy evoke distress, as romantic connections are fundamental to psychological health and life satisfaction [49, 50]. The intensity of this sadness can indicate the depth of emotional longing for romantic involvement [51]. The third item (I feel left out when I see other couples expressing affection) assesses social comparison and perceived exclusion from romantic experiences. Observing others in affectionate relationships can intensify feelings of loneliness and inadequacy, reinforcing the psychological impact of being romantically unattached [52]. Online social networks also increase such loneliness [37, 38]. This aspect of romantic loneliness is particularly significant, as it can contribute to decreased self-esteem and increased longing for romantic relationships [39, 40, 50]. The fourth item (I feel like others are having better romantic experiences than I am) reflects an individual’s perception of being at a disadvantage in romantic fulfillment compared with peers. Social comparison theory suggests that perceiving oneself as less romantically successful can heighten feelings of loneliness and dissatisfaction [41]. This item effectively captures the cognitive dimension of romantic loneliness by highlighting unfulfilled expectations and self-perceived inadequacy. Individuals evaluate their own experiences and attributes by comparing themselves to others, and unfavorable comparisons can lead to negative emotional states [42, 43]. In the context of romantic relationships, seeing others in fulfilling romantic partnerships may intensify feelings of romantic loneliness and dissatisfaction with one’s own relational status [41]. Research further indicates that upward social comparisons in the romantic domain, such as observing peers in seemingly happy relationships, can exacerbate feelings of exclusion and lower self-esteem, reinforcing romantic loneliness [44].

Following the successful development and psychometric validation of the RomLon Scale, romantic loneliness may now be operationally defined as an emotional state characterized by the perceived absence of meaningful romantic connection and an unfulfilled desire for intimacy. It is a present-moment experience rooted in four interrelated psychological themes: (1) Romantic Desires, reflected in persistent cognitive preoccupation with romantic thoughts and an internal longing for emotional closeness; (2) Feelings of Sadness, emerging from the emotional pain and distress associated with being romantically alone; (3) Exclusion and Comparison, wherein individuals feel left out when witnessing others’ romantic affection, highlighting a sense of social disconnection; and (4) Envy and Inadequacy, which involve upward social comparisons and feelings of romantic inferiority relative to peers. Romantic loneliness is conceptually distinct from general loneliness, fear of being single, or relationship anxiety, as it specifically addresses emotional and cognitive dissatisfaction within the romantic domain. It also excludes instances of intentional or selectively chosen romantic solitude, wherein individuals are single by choice and do not experience associated distress. Thus, the RomLon Scale captures a unique construct that reflects the emotional, cognitive, and social dimensions of romantic unfulfillment.

The RomLon scale was significantly correlated with the UCLA loneliness scale, confirming convergent validity. Divergent validity was established via a strong negative correlation with the psychosocial life satisfaction scale. The RomLon scale also exhibited strong predictive power for hopelessness and sexual distress. The findings establish the RomLon scale as a psychometrically robust measure of romantic loneliness. Its strong reliability, factor structure, and predictive validity underscore its utility in assessing romantic loneliness and its impact on psychosocial health.

The findings of this study highlight the growing prevalence of romantic loneliness in the digital age, particularly within the contexts of online dating, hookup culture, and marital relationships. The significant positive correlation between romantic loneliness and age suggests that older individuals tend to experience more romantic loneliness. Older individuals possess greater emotional [76], social [77], and sexual intelligence [78], which enhances their capacity for introspection and a deeper evaluation of their personal and subjective satisfaction. Research has indicated that as individuals age, their opportunities for romantic engagement may decline due to factors such as reduced social networks, life transitions, and shifting priorities [79]. Older individuals may have an increasing need for romance due to evolving emotional and psychological needs, a desire for companionship, and the maintenance of life satisfaction. As people age, they often experience shifts in social roles, changes in family dynamics, and increased vulnerability to loneliness, all of which can intensify their need for meaningful romantic connections [80, 81]. Socioemotional selectivity theory posits that as individuals grow older, they prioritize emotionally fulfilling relationships over superficial social interactions, making romantic bonds particularly significant later in life [80]. Furthermore, research suggests that romantic relationships contribute to psychological wellbeing, emotional security, and overall life satisfaction in older adults [82]. Even in later years, intimacy remains a fundamental human need, and older individuals who lack romantic companionship may experience emotional distress and a diminished sense of purpose [83]. Additionally, studies have shown that older adults who maintain romantic relationships report better mental health and lower levels of depression, reinforcing the argument that romance remains a crucial aspect of well-being across the lifespan [84].

The current study revealed a significant positive correlation between romantic loneliness and education, which suggests that highly educated individuals tend to experience more romantic loneliness. Highly educated individuals often have elevated expectations for romantic relationships, which, if they are unmet, may contribute to heightened romantic loneliness [85]. Education fosters critical thinking, self-awareness, and an expanded worldview [86], which may lead individuals to seek partners who align with their intellectual and emotional needs [85]. As a result, when these expectations are unmet, they may experience heightened romantic loneliness due to difficulty in finding a suitable partner who meets their standards for companionship, emotional connection, and intellectual compatibility [87]. Moreover, research suggests that highly educated individuals may prioritize career advancement and personal achievements over romantic pursuits, leading to delayed relationships or a reduced pool of compatible partners [3]. This delay or difficulty in establishing romantic relationships may increase their vulnerability to romantic loneliness, as they may struggle to find partners who share their values and aspirations [88]. Additionally, education may heighten individualistic tendencies, making it challenging for some highly educated individuals to compromise or adjust their expectations in romantic relationships, further exacerbating feelings of loneliness [89].

The findings of the current study suggest that men, although by moderate effect size, experience higher levels of romantic loneliness. Research suggests that men may experience higher levels of loneliness due to a combination of social, psychological, and cultural factors. Traditional gender roles often discourage men from expressing vulnerability and seeking emotional support, leading to social isolation and unmet emotional needs [55, 90]. Unlike women, who tend to engage in more emotionally expressive and supportive social networks, men’s relationships are often structured around shared activities rather than deep emotional connections [91]. This lack of emotional intimacy can contribute to feelings of loneliness, particularly when romantic or close friendships are absent. Furthermore, masculinity norms emphasizing self-reliance and emotional stoicism can hinder men’s willingness to seek help for emotional distress, further exacerbating their loneliness [92]. Studies also suggest that men’s social circles shrink with age, as they are less likely than women are to maintain lifelong friendships or actively build new friendships, increasing their risk of loneliness later in life [93]. Additionally, research on romantic relationships highlights that men tend to rely more heavily on their romantic partners for emotional support than women do [94]. Consequently, men who are single, divorced, or widowed are more prone to experiencing intense loneliness, as they may lack alternative sources of emotional support [95, 96].

The findings of the current study also suggest that married individuals, although by moderate effect size, experience higher levels of romantic loneliness. Research on marital satisfaction has shown that being in a relationship does not necessarily prevent loneliness, as married individuals who experience emotional disconnection or dissatisfaction in their relationship may report greater loneliness than their unmarried counterparts [97–99]. The presence of unmet emotional needs within marriage can lead to a paradoxical increase in romantic loneliness, despite being in a committed relationship [100]. These findings contribute to the growing body of literature highlighting the complex interplay between demographic factors and romantic loneliness, emphasizing the need for further research to explore the underlying mechanisms driving these associations.

### Implications

The RomLon scale has significant implications for both research and clinical practice. As a psychometrically sound instrument, it provides a targeted assessment of romantic loneliness, distinguishing it from general loneliness scales that fail to capture the emotional and psychological distress stemming specifically from the absence of romantic intimacy. This scale can aid researchers in exploring the antecedents and consequences of romantic loneliness, such as its links to mental health issues such as depression, anxiety, and hopelessness. Clinicians and counselors can use the RomLon scale to identify individuals at risk of experiencing romantic loneliness and develop interventions that enhance emotional resilience, relationship readiness, and social support. Future research could focus on developing and testing interventions aimed at reducing romantic loneliness. Furthermore, its brevity and cultural sensitivity make it a practical tool for use in similar cultures, ensuring that individuals can express their experiences without discomfort. The RomLon scale also paves the way for future studies examining the impact of romantic loneliness on life satisfaction, relational wellbeing, and overall psychosocial health.

## Conclusion

The development and validation of the RomLon scale address a critical gap in psychological assessment by providing a reliable and culturally sensitive tool to measure romantic loneliness. The RomLon-scale is a promising tool for assessing romantic loneliness, with strong psychometric properties and clear implications for research and clinical practice.

Unlike general loneliness scales, the RomLon scale captures the distinct emotional distress associated with the absence of romantic intimacy, making it a valuable instrument for both researchers and practitioners. The findings of the present study reinforce the importance of romantic loneliness as a unique construct with implications for mental health, life satisfaction, and relational well-being. Given its strong psychometric properties, this scale can facilitate future research on the predictors and consequences of romantic loneliness while also assisting mental health professionals in designing targeted interventions.

## Data Availability

Availability of data and materials: Data associated with this paper can be produced upon request from the corresponding author.

## Abbreviations

ANOVA: Analysis of variance
BTS: Bartlett’s test of sphericity
CFA: Confirmatory Factor Analysis
CFI: Comparative Fit Index
EFA: Exploratory Factor Analysis
GFI: Goodness-of-fit index
HTMT: Heterotrait–Monotrait ratio
ICC: Interclass correlation
IFI: Bollen’s Incremental Fit Index
KMO: Kaiser–Meyer–Olkin measure of sampling adequacy
MANCOVA: Multivariate analysis of covariance
MFI: McDonald’s fit index
NFI: Bentler–Bonett Normed Fit Index
NNFI: Bentler–Bonett Nonnormed Fit Index
PNFI: Parsimony Normed Fit Index
RFI: Bollen’s relative fit index
RomLon: Romantic Loneliness Scale
RMSEA: Root mean square error of approximation
RNI: Relative Noncentrality Index
SRMR: Standardized root mean square residual
TLI: Tucker‒Lewis Index
